# A Rare Cause of Lymphadenopathy in a Young Hispanic Female: Unmasking Recurrent Nodal Rosai-Dorfman Disease

**DOI:** 10.7759/cureus.39131

**Published:** 2023-05-17

**Authors:** Blesset Alexander, Alfarooq Alshaikhli, Mery Bartl, Yilen K Ng-Wong, Emilia C Dulgheru

**Affiliations:** 1 Internal Medicine, University of Texas Rio Grande Valley, Edinburg, USA; 2 Internal Medicine, DHR Health, Edinburg, USA; 3 Rheumatology, University of Texas Rio Grande Valley, Edinburg, USA

**Keywords:** 6-mercaptopurine, hispanic female, steroid-sparing agent, sinus histiocytosis with massive lymphadenopathy, recurrent rosai dorfman disease

## Abstract

Rosai-Dorfman disease (RDD) is a rare histiocytic disorder that can present with painless bilateral symmetrical cervical lymphadenopathy, mimicking lymphomas. RDD is characterized by excessive tissue infiltration by dendritic cells, macrophages, or monocyte-derived cells, with a histopathologic diagnosis based on the presence of CD68+, CD163+, and S100+ histiocytes, which differentiate it from other histiocytic neoplasms. In this case report, we present a young Hispanic female with recurrent subcutaneous growths and lymphadenopathy, initially thought to be lymphoma, who was diagnosed with RDD after a significant diagnostic workup. Treatment initially consisted of surgical excision; however, due to recurrence, the patient was successfully treated with corticosteroids and a steroid-sparing agent, 6-mercaptopurine, with significant improvement in symptoms. RDD should be considered a differential diagnosis for patients with cervical lymphadenopathy, and an interdisciplinary approach is essential to managing this rare disorder effectively. The report highlights the need for an interdisciplinary approach to managing this rare disorder effectively and underscores the importance of multimodal treatment in disease suppression. As a rare disease with slow advancement of defined guidelines for diagnostic and treatment strategies, this case report adds to the existing literature on RDD.

## Introduction

Rosai-Dorfman disease (RDD), also known as Rosai-Dorfman-Destombes syndrome, is a rare histiocytosis disorder of sinus histiocytosis with massive lymphadenopathy with 1:200000 prevalence and estimated 100 new cases reported yearly in the United States [1]. In general, histiocytosis is characterized by excessive tissue infiltration by histiocytes (dendritic cells), macrophages, or monocyte-derived cells. Histopathologic diagnostic characteristics of RDD include CD68+, CD163+, S100+, factor XIIIa+/−, CD1a−, Langerin−, and BRAF V600E− histiocytes, which differentiates it from other histiocytic neoplasms, including Erdheim-Chester disease and Langerhans cell histiocytosis [2]. Common clinical presentation of RDD consists of painless bilateral symmetrical cervical lymphadenopathy with or without B symptoms like fever, fatigue, weight loss, and leukocytosis. This typical clinical presentation mimics that of lymphomas, so it is important to keep RDD on the differential as it has a more favorable prognosis and response to treatment [2].

We present a case of a young Hispanic female who presented with recurrent subcutaneous growths in the neck and lymphadenopathy, which was initially believed to be lymphoma. After a significant diagnostic workup, the patient was diagnosed with Rosai-Dorfman syndrome. Treatment initially consisted of surgical excision; however, due to recurrence, the patient was successfully treated with corticosteroids and a steroid-sparing agent with significant improvement of symptoms. This case report highlights the importance of multimodal treatment in disease suppression in patients with RDD.

## Case presentation

**Figure 1:**
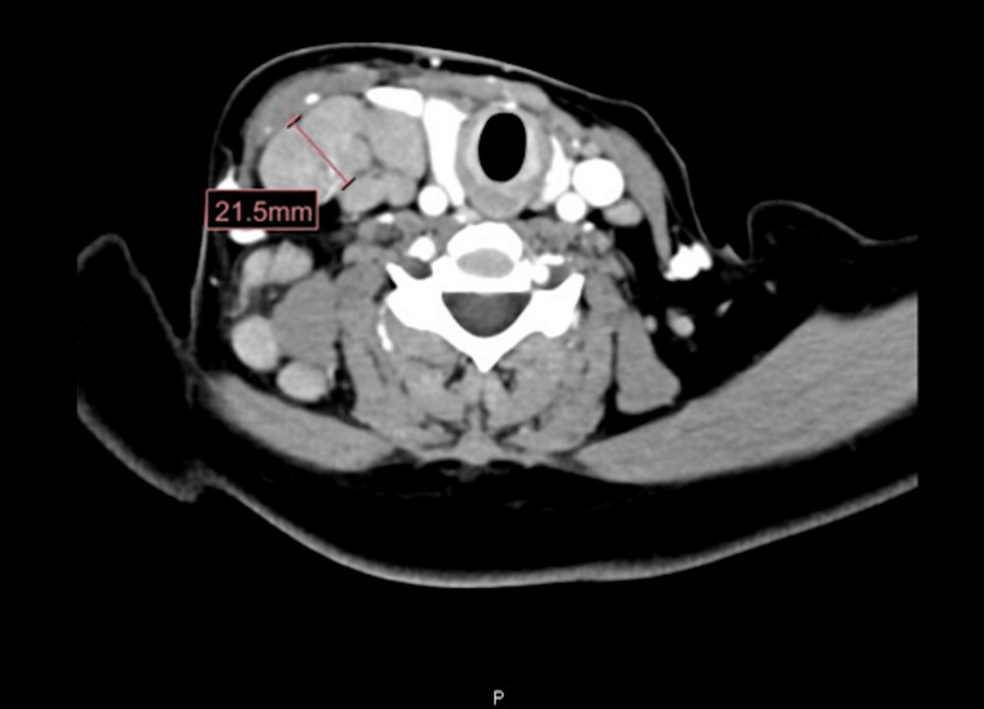
CT neck with contrast (axial view) showing severe bilateral neck lymphadenopathy most prominent on the right side at the jugulodigastric region level (2A and 2B), uplifting the right sternocleidomastoid muscle CT, computerized tomography

**Figure 2:**
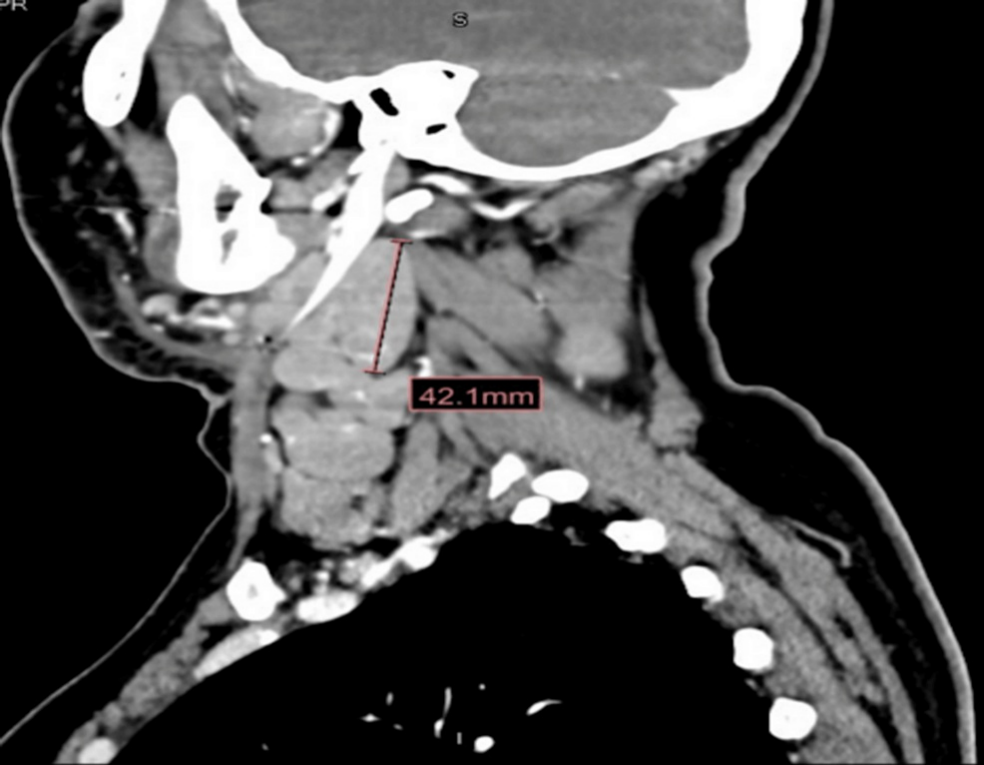
CT neck with contrast (coronal view) showing severe bilateral neck lymphadenopathy most prominent on the right side measuring up to 4.2 cm at the jugulodigastric region level (2A and 2B), uplifting the right sternocleidomastoid muscle CT, computerized tomography

The patient is a 40-year-old Hispanic lady who presented to the rheumatology clinic referred by her otolaryngologist (ENT) for complaints of recurrent right-sided neck mass growth. The first time she noticed the growth was in 2010 when she felt a small bump growing on the right side of her neck. At that time, she had a surgical excision in Mexico and had a repeat excision due to recurrence a few years later. The patient reported that her excisional biopsies were benign, requiring no further treatment or follow-up. However, 10 years later, she started noticing regrowth of the mass at the same site with a progressive increase in size. Because of the rapid progression of the mass, the diagnosis of lymphoma was entertained, and the patient was subsequently referred to an ENT. Computerized tomography (CT) of the chest/neck with contrast revealed asymmetric left axillary adenopathy, largest in the left deep axillary region extending slightly subpectoral, measuring up to 3.9 × 1.6 cm, and severe bilateral neck lymphadenopathy most prominent on the right (Figures 1, 2).

**Figure 3:**
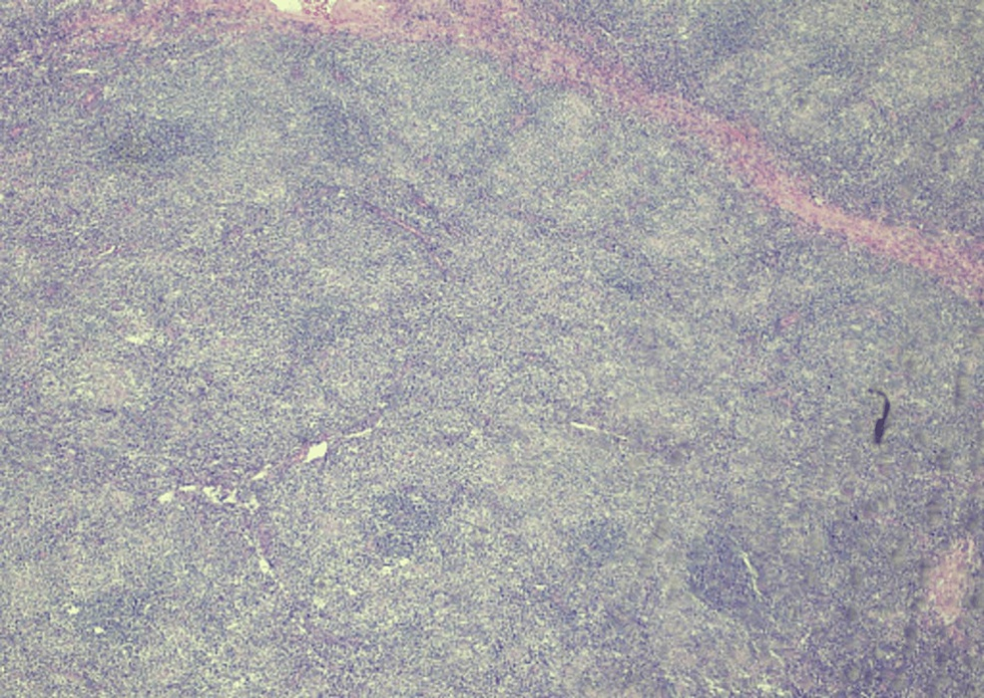
Microscopic examination of an H&E stained section of a nodal RDD sample, with low magnification (4×) revealing expanded lymph node tissue with nodal architecture disruption H&E, hematoxylin and eosin stain; RDD, Rosai-Dorfman disease

**Figure 4:**
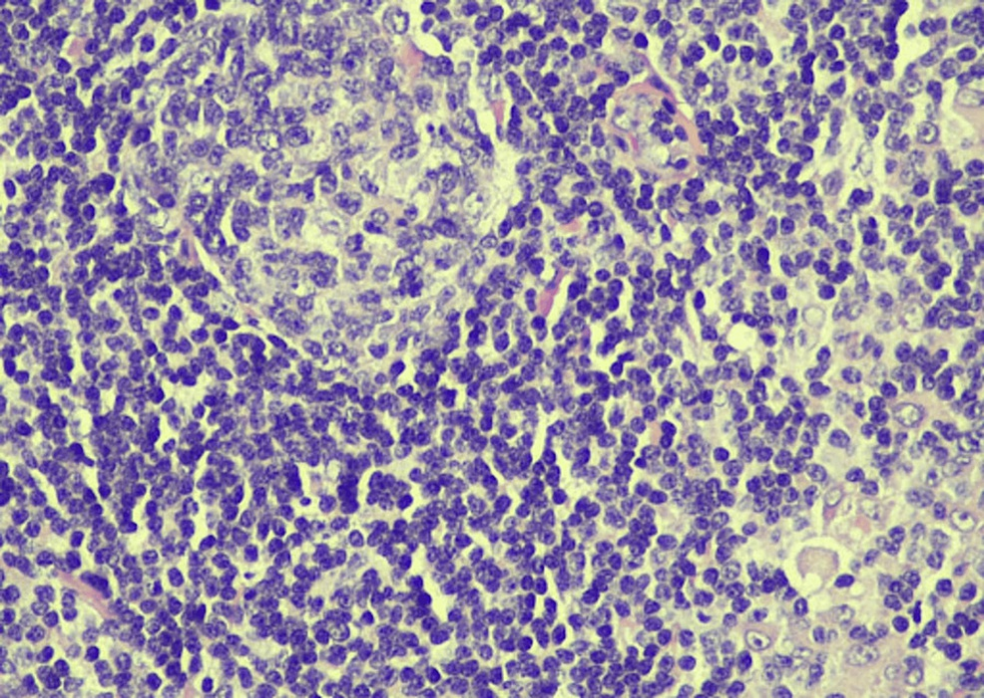
Microscopic examination of an H&E stained section of a nodal RDD sample, with higher magnification (40×) revealing characteristic features, including the presence of foamy histiocytes with large nuclei, emperipolesis, and mixed inflammatory infiltrate Of note, these cells were S100+, CD68+, and CD163+ and negative for CD1a and BRAF V600E by immunohistochemistry, which is typical for RDD H&E, hematoxylin and eosin stain; RDD, Rosai-Dorfman disease

**Figure 5:**
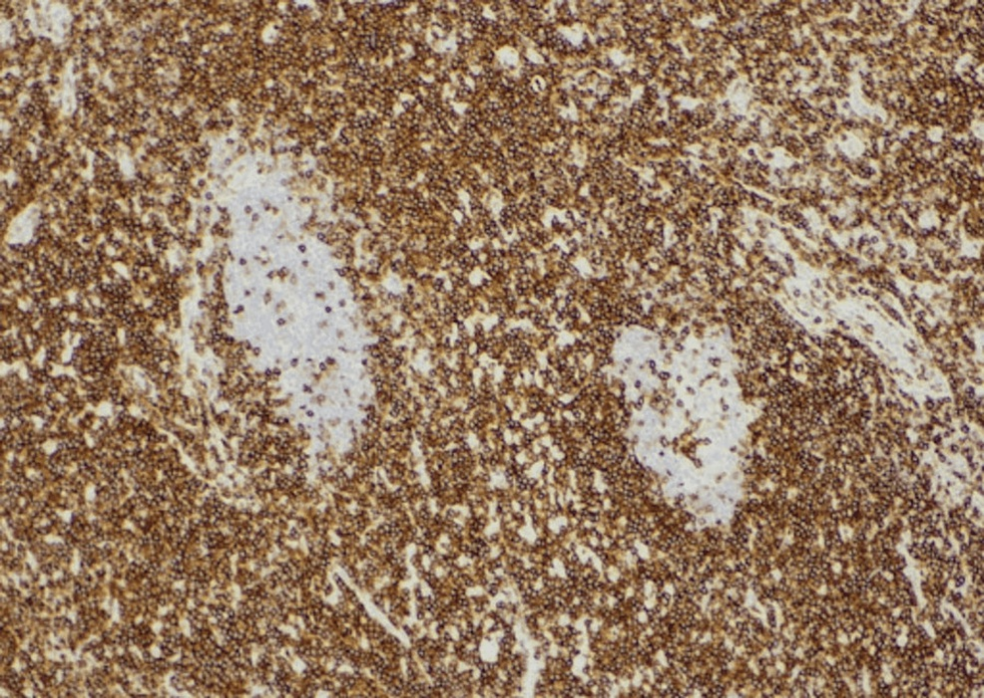
CD5 staining highlighting the histiocytes' cytoplasm, indicating their histiocytic nature and helping to confirm the RDD diagnosis RDD, Rosai-Dorfman disease

**Figure 6:**
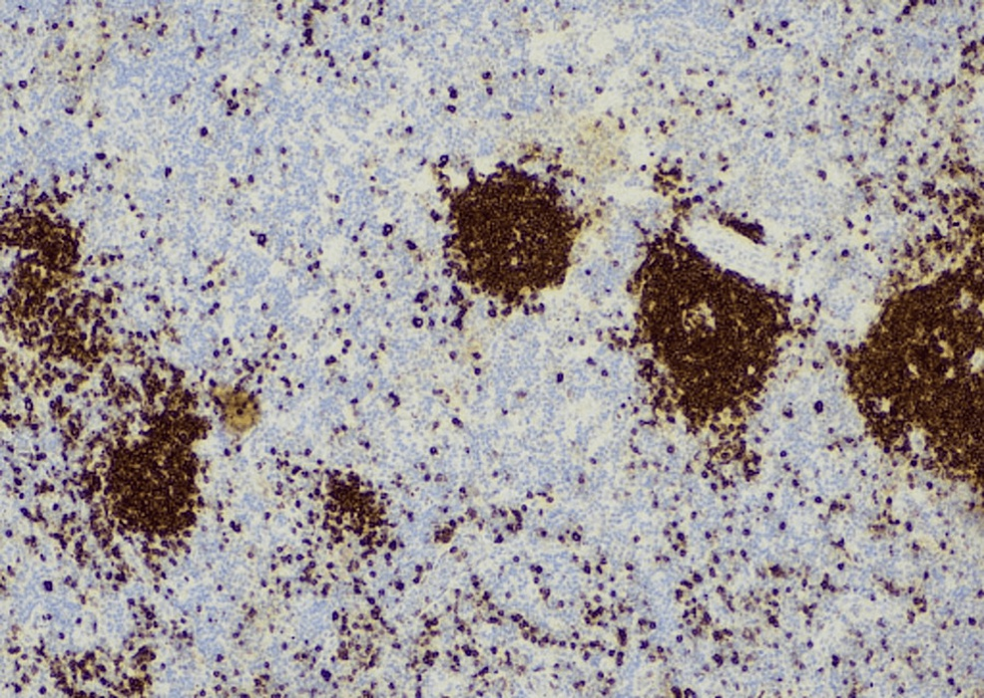
CD20 staining highlighting the presence of B cell clusters or aggregates within the histiocytic infiltrate

She was evaluated and underwent an ultrasound of soft tissue neck/thyroid with fine needle aspiration (FNA), and pathology results revealed a benign heterogeneous population of small- to medium-sized lymphocytes, without findings of malignant cells. The patient underwent an excisional biopsy of the enlarged right lymph node to completely rule out Hodgkin’s lymphoma. Her biopsy reports of lymph node sections showed a paracortical expansion associated with sinus histiocytosis. An immunohistochemical panel of stains showed CD45 that highlights the lymphocytic milieu of the lymph node; B cells highlighted in a cortical and follicular pattern by CD20, PAX5, BOB1, and OCT2; a minor subset of B cells highlighted by MUM1 in a paracortical distribution; T cells highlighted in a paracortical distribution by CD3, CD5, and CD43; sinus histiocytes highlighted by BCL1 (cyclin D1), CD68, CD163, S100, and OCT2; lymphocytes highlighted by Ki-67 in a paracortical distribution pattern; and CD15 and CD30 highlighted nonspecifically in a paracortical pattern and negative for AE1/AE3, EMA, and CD1a (Figures 3-6).

**Figure 7:**
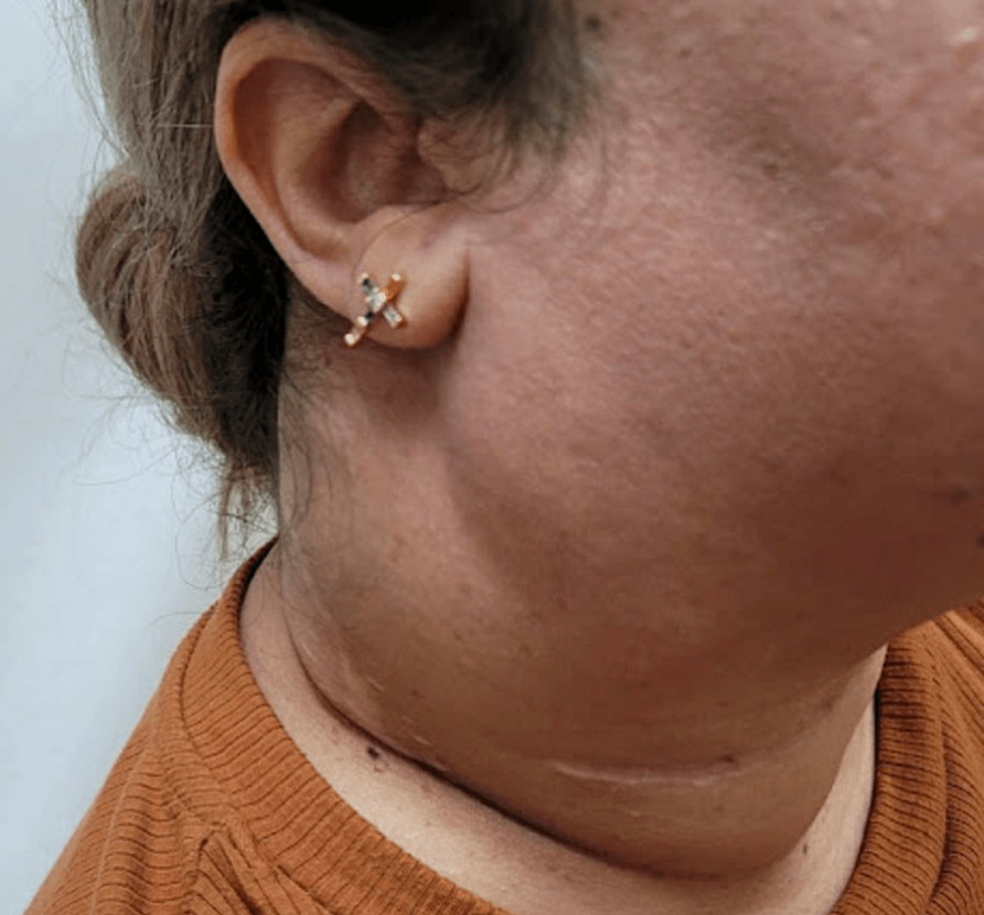
Lateral view of the right-sided neck mass

Flow cytometry came negative for abnormal or neoplastic lymphoid populations, and no malignancy was identified. Overall, the interpretation combined with morphology came consistent with RDD (sinus histiocytosis with massive lymphadenopathy), and she was diagnosed with RDD. The case was discussed in the tumor board, the decision was taken for the patient to be started on oral steroid prednisone, and the patient was referred to rheumatology for medical treatment in the setting of recurrent tumor growth despite excisions. While awaiting her rheumatology appointment, she was noted to have an improvement in size with steroids and was tapered down the regimen; however, she developed rapid regrowth of the mass. She had no associated symptoms, fever, chills, visual changes, ear discomfort, dysphagia, night sweats, weight loss, joint pain, Raynaud's phenomenon, photosensitivity, malar rash, discoid lesions, vasculitic lesions, livedo reticularis, oral ulcers, sicca, and subcutaneous nodules. Her physical examination revealed vital signs within normal limits and a 7 × 5 cm mass at the right cervical region extending to the postauricular region (Figure 7).

In the setting of her long-standing history, recurrent mass after excision, and potential of local compression and local complications considering the location, she was considered for further treatment with immunosuppression followed by a chemotherapeutic agent. She was reinitiated on oral steroids such as prednisone 20 mg twice daily, with which she improved, and her follow-up visit showed improvement in jaw swelling. Detailed lab work included complete blood count, antinuclear antibody by immunofluorescence assay, serum protein electrophoresis, erythrocyte sedimentation rate, C-reactive panel, comprehensive metabolic profile, angiotensin-converting enzyme level, microcytic anemia, and low vitamin D levels. The lab results of vitamin D level for leukocytosis came back remarkable. IgG4 levels came back normal, and IgG4/IgG ratio was considerably low. The lab results of BRAF V600E, NRAS, KRAS, ARAF, PIK3CA, MAP2K1, and ALK, which were ordered to evaluate whether she was a candidate for targeted therapy, came back negative. Her thiopurine S-methyltransferase (TPMT) activity came back normal, and she was seen with an excellent clinical response to the steroids in two weeks. She was started on a tapering regimen for steroids along with steroid-sparing agent 6-mercaptopurine 50 mg daily, as the total duration of treatment is 12-16 weeks.

## Discussion

RDD is a rare medical disorder with a prevalence of 1:200000 initially described in 1965 by a French pathologist, Pierre-Paul Louis Lucien Destombes, and four years later by Rosai and Dorfman in 1969, as sinus histiocytosis with massive lymphadenopathy [1]. From the time of initial classification, many changes have come; this is the reason why the Histiocyte Society has come up with the latest revised classification for histiocytic disorders, where they are classified into five groups of diseases including Langerhans-related, cutaneous and mucocutaneous, malignant histiocytosis, RDD and hemophagocytic lymphohistiocytosis, and macrophage activation syndrome [2]. The RDD has been further classified into familial, classical (nodal), extranodal, neoplasia-associated, and immune disease-associated. The nodal type is the most common, with the classic presentation being bilateral painless cervical lymphadenopathy, even though other lymph nodes can also be affected. Extranodal disease is the next common type involving the skin, nasal cavity, bone, soft tissue, and retro-orbital tissue, with around 43% of documented cases [2]. The cutaneous presentation usually consists of deep red papules, plaques, or nodules [3].

Our patient’s presentation was consistent with the nodal disease. The etiology of RDD is still unclear; it is reported to be associated with some autoimmune diseases, various viral infections, and Hodgkin’s lymphoma, which can precede or follow the diagnosis as initiation triggers. There have been many advancements in research studies in other histiocytic disorders; however, clinical spectrum and treatment methods have yet to be well-defined for RDD due to the rarity of the disease. The latest studies have identified NRAS, KRAS, MAP2K1, and ARAF mutations in patients with features of RDD; however, further research studies need to be done [4]. It is crucial to distinguish RDD from lymphoma as clinical presentations are similar; however, treatment varies widely [5]. Our case initially was suspected as lymphoma; however, surgical biopsy proved it to be typical for RDD, and flow cytometry came back revealing no malignant cells, which helped us differentiate it from lymphoma.

A comprehensive evaluation for RDD should also include assessing IgG4 levels and the IgG4/IgG ratio, since some RDD patients have elevated serum IgG4 levels and the presence of IgG4-positive plasma cells in lymph nodes and tissues [6]. Diagnostic criteria for IgG4-related disease involve the observation of over 100 cells per high-powered field in lymph nodes, with an IgG4-positive to IgG-positive plasma cell ratio exceeding 40%, which is atypical for RDD [6]. Additionally, RDD is characterized by histopathological findings of S100+ histiocytes displaying emperipolesis, while fibrosis is a common feature of IgG4-related disease [6]. In our patient’s case, normal serum IgG4 levels and the IgG4/IgG ratio, along with distinct histopathological characteristics, clearly indicated RDD, rendering IgG4 staining unnecessary.

In the literature, it can be seen that RDD is a benign but progressive disease, with 20-50% of nodal presentations having spontaneous remissions. Treatment strategies are usually individualized on a case-to-case basis. Surgical resection is usually preferred for unifocal disease or for debulking in case of complicated presentations causing compressive symptoms [4]. In our patient, surgical resection and excisional biopsy were done for her initial presentation as subcutaneous swelling since it was a unifocal disease and also for diagnostic purposes. On the third recurrence, she again presented with a local mass extending to lymph nodes. To establish a diagnosis, she first underwent FNA cytology (FNAC) followed by surgical biopsy and lymph node dissection. While excisional biopsy is considered a superior diagnostic method, FNAC has been studied as a quick, effective, and relatively inexpensive approach for evaluating superficial masses, particularly in the neck area [7]. FNAC, in this case, was benign and only showed a benign heterogeneous population of small- to medium-sized lymphocytes, which was negative for malignant cells, and there was a concern for malignant lymphoma. However, her flow cytometry and biopsy cytology helped to rule out lymphoma and other histiocytic disorders and confirm RDD.

This brings us to the discussion on treatment options when it comes to recurrence. Immunosuppressive therapy with steroids has been shown effective in a few studies for size reduction and symptom management. However, the duration and dose of therapy are not well defined. In a study done among 64 patients in a tertiary referral center over 23 years (1994-2017) by Goyal et al., cladribine showed a high overall response rate [3]. Other agents with sustained clinical response were 6-mercaptopurine, azathioprine, methotrexate, and, in a few cases, rituximab [8]. Further literature review shows that various studies have used these agents in different combinations in refractory cases, leading to significant improvement [3,9]. There have been breakthrough advancements in the treatment of other refractory histiocytic diseases through next-generation genomic sequencing and targeted therapy. In a study done with 20 RDD specimens by Garces et al. in 2017, it was interesting to see that 33% of archive RDD specimens showed mutually exclusive recurrent somatic KRAS and MAP2K1 mutations [10]. In our case, the patient had genomic testing sent for BRAF V600E, NRAS, KRAS, ARAF, PIK3CA, MAP2K1, and ALK; however, no mutation was detected. She did not want to be on contraceptives; hence, she was started on 6-mercaptopurine after checking her TPMT activity, which came back normal. There have been many propositions on the duration of therapy; however, based on the consensus recommendations published in 2018 by Abla et al., a total duration of six to 12 months of systemic therapy with monitoring for an initial response of around four months is considered a reasonable approach [5]. RDD presentation has a wide clinical spectrum, and mortality has been reported in the literature. Currently, chemotherapy has been reserved for resistant or refractory cases or those with a life-threatening initial presentation. Research studies have been ongoing, and one such study can be found in the US National Library of Medicine, clinical trials.gov.

## Conclusions

In conclusion, this case report on RDD emphasizes the importance of a diagnostic strategy for RDD, differentiates it from malignant lymphoma, and discusses available treatment options for refractory or recurrent presentations.

RDD is a rare histiocytic disorder characterized by excessive tissue infiltration by dendritic cells, macrophages, or monocyte-derived cells. The presented case highlights the challenges of diagnosing RDD, the initial misdiagnosis, and the successful treatment with corticosteroids and 6-mercaptopurine after surgical excision. Considering the rarity of the disease and slower advancement regarding defined guidelines for diagnostic and treatment strategies, we hope the case report can add to the existing literature.

## References

[1] Nguyen PX, Nguyen NV, Le TD (2021). Spinal extranodal Rosai-Dorfman disease: a case report and literature review. Int J Surg Case Rep.

[2] Emile JF, Abla O, Fraitag S (2016). Revised classification of histiocytoses and neoplasms of the macrophage-dendritic cell lineages. Blood.

[3] Goyal G, Ravindran A, Young JR (2020). Clinicopathological features, treatment approaches, and outcomes in Rosai-Dorfman disease. Haematologica.

[4] Magableh HM, Jaber HD, Magableh AM, Alrabiah MA, Dahhan AF, Azzam AZ, Amin T (2023). Rosai-Dorfman disease: case series and literature review. Cureus.

[5] Abla O, Jacobsen E, Picarsic J (2018). Consensus recommendations for the diagnosis and clinical management of Rosai-Dorfman-Destombes disease. Blood.

[6] Chen LYC, Slack GW, Carruthers MN (2021). IgG4-related disease and Rosai-Dorfman-Destombes disease. Lancet.

[7] Hashmi AA, Naz S, Ahmed O, Yaqeen SR, Irfan M, Kamal A, Faridi N (2020). Utility of fine needle aspiration cytology in the evaluation of lymphadenopathy. Cureus.

[8] Lu D, Estalilla OC, Manning JT Jr, Medeiros LJ (2000). Sinus histiocytosis with massive lymphadenopathy and malignant lymphoma involving the same lymph node: a report of four cases and review of the literature. Mod Pathol.

[9] Michaeli O, Elassa M, Williams R, Baltazar G (2019). Recurrent cutaneous Rosai-Dorfman disease. Cureus.

[10] Garces S, Medeiros LJ, Patel KP (2017). Mutually exclusive recurrent KRAS and MAP2K1 mutations in Rosai-Dorfman disease. Mod Pathol.

